# Different mechanisms underpin the decline in growth of anchovies and sardines of the Bay of Biscay

**DOI:** 10.1111/eva.13564

**Published:** 2023-08-07

**Authors:** Andy Boëns, Bruno Ernande, Pierre Petitgas, Christophe Lebigre

**Affiliations:** ^1^ Ifremer EMH, Centre Atlantique Nantes France; ^2^ Université de Montpellier – Campus Triolet – Place E. Bataillon Montpellier France; ^3^ Ifremer Fisheries Science and Technology Unit, Centre Bretagne Plouzané France

**Keywords:** fisheries‐induced evolution, growth, growth compensation, heritability, selection, small pelagic fish

## Abstract

Declines in individuals' growth in exploited fish species are generally attributed to evolutionary consequences of size‐selective fishing or to plastic responses due to constraints set by changing environmental conditions dampening individuals' growth. However, other processes such as growth compensation and non‐directional selection can occur and their importance on the overall phenotypic response of exploited populations has largely been ignored. Using otolith growth data collected in European anchovy and sardine of the Bay of Biscay (18 cohorts from 2000 to 2018), we parameterized the breeder's equation to determine whether declines in size‐at‐age in these species were due to an adaptive response (i.e. related to directional or non‐directional selection differentials within parental cohorts) or a plastic response (i.e. related to changes in environmental). We found that growth at age‐0 in anchovy declined between parents and their offspring when biomass increased and the selective disappearance of large individuals was high in parents. Therefore, an adaptive response probably occurred in years with high fishing effort and the large increase in biomass after the collapse of this stock maintained this adaptive response subsequently. In sardine offspring, higher growth at age‐0 was associated with increasing biomass between parents and offspring, suggesting a plastic response to a bottom‐up process (i.e. a change in food quantity or quality). Parental cohorts in which selection favoured individuals with high growth compensation produced offspring high catch up growth rates, which may explain the smaller decline in growth in sardine relative to anchovy. Finally, on non‐directional selection differentials were not significantly related to the changes in growth at age‐0 and growth compensation at age‐1 in both species. Although anchovy and sardine have similar ecologies, the mechanisms underlying the declines in their growth are clearly different. The consequences of the exploitation of natural populations could be long lasting if density‐dependent processes follow adaptive changes.

## 1 INTRODUCTION

The evolutionary potential of natural populations determines their ability to respond to the selection pressures exerted by environmental and trophic changes and anthropogenic disturbances (Crozier & Hutchings, 2014; Heino & Rune Godø, 2002; Johnson et al., 2010; Malvezzi et al., 2015). Indeed, the adaptive response of populations to past events (Carlson & Seamons, 2008; Johnson et al., 2012) can strongly constraint their ability to deal with new selection pressures and/or changes in the magnitude or direction of selection in space or time. This effect is particularly acute when ecological processes such as density‐dependent processes occur following an adaptive response (Hanski, 2011; Merilä & Hendry, 2014; Uusi‐Heikkilä et al., 2015). As population's growth rates are often negatively density‐dependent, the declines in populations' size following selection (and an adaptive response) can strongly influence populations' ability to recover (Dunlop et al., 2015). Therefore, it is crucial to understand the interplay between populations' adaptive responses and the changes in their dynamics over time to identify the constraints shaping their phenotypic characteristics.

A decrease in individuals' size‐at‐age has been observed in many exploited fish populations (Baudron et al., 2014), which may be associated with a decrease in growth rate, an earlier maturity and/or an increased energy allocation to reproduction. Changes in growth rates over time are often associated with size selective fishing which, by increasing the mortality rate of large individuals, leads to populations' evolutionary response and (depending on their reaction norms) to an increase in the frequency of smaller individuals, with earlier maturation and a decrease in reproductive success (Enberg et al., 2009; Heino et al., 2015; Hutchings & Fraser, 2008; Nusslé et al., 2009; Swain et al., 2007). But in addition to fisheries‐induced evolutionary changes, environmental changes can also lead to a decline in individuals' growth rate either through a plastic response resulting in the reallocation of resources towards reproduction (Heino et al., 2008; Saraux et al., 2019) and/or the selective mortality of rapidly growing individuals' due to, for instance, their higher somatic maintenance costs or trade‐offs with survival (e.g. Dmitriew, 2011; Metcalfe & Monaghan, 2003; Ohlberger, 2013). It is, therefore, necessary to quantify the selection differentials acting on growth and estimate its heritability to determine whether phenotypic plasticity or evolutionary changes explain the declines in size‐at‐age (Reusch, 2014), that is, the proportion of phenotypic variability explained by genetic (additive) variance (Crozier & Hutchings, 2014; Franks et al., 2014; Merilä & Hendry, 2014). Heritability estimates for fish growth range from 0.1 to 0.5 (Carlson & Seamons, 2008; Garcia de Leaniz et al., 2007; Law, 2000; Nusslé et al., 2009; Swain et al., 2007; Thériault et al., 2007) making this trait prone to evolutionary changes. The focus on fisheries‐induced evolution has led us to primarily focus on directional selection, while the overall evolutionary response of individuals' growth may also depend on the magnitude and direction of non‐directional selections (i.e. balancing or diversifying selection; Hansen, 1997; Duda et al., 2002). While balancing selection is commonly reported and can act as a stabilizing force, diversifying selection can lead to an increase in the variance in phenotypes and drive the evolution of alternative strategies and help maintaining a high variance in fitness‐related traits (Garcia et al., 2012; Law et al., 2016; Law & Plank, 2018). Therefore, considering species response to directional selection (largely assumed to be driven by selective fishing) and non‐directional selection (primarily driven by environmental factors) is required to fully understand the causes and mechanisms underlying declines in body size of exploited marine populations.

Two methods have been mainly used to study evolutionary changes in exploited marine populations: reaction norms and parameterizations of the breeder's equation. Reaction norms explain the range of any phenotypes expressed by a genotype along an environmental gradient (Dieckmann & Heino, 2007; Heino et al., 2002; Hutchings & Fraser, 2008). Parameterizations of the breeder's equation are based on time series of phenotypic traits to estimate differences between parents and offspring (response to selection), selection differentials on these traits (within parental generations) and differences in environmental conditions between parents and offspring also used to estimate the additive variance in a trait (Hendry, 2016; Lynch et al., 1998). Swain et al. (2007) applied this method to fisheries data to show an evolutionary response in an exploited marine fish population. However, the environmental factors considered are often limited to those easily measured during fisheries surveys (temperature and abundance), while others such as food quantity might be even more important but difficult to estimate (Boëns et al., 2021; Daufresne et al., 2009; Forster et al., 2011). Moreover, non‐directional selection has largely been ignored in these studies, while this selection can be very common (Johnson et al., 2012, 2014). Finally, many studies have focused on size‐at‐age, while processes such as growth compensation (another name is catch‐up growth), which is the ability of individuals to recover from a period of limited growth (Ali et al., 2003; Dmitriew, 2011; Metcalfe & Monaghan, 2001; Wright et al., 2007), can also significantly influence changes in size with age. Growth compensation represents the ability of a fish with a relatively slow growth rate during the first year (from age 0 to age 1) to undergo rapid growth during the second year (from age 1 to age 2). In species with nonlinear asymptotic growth, any delay in the onset of growth will obviously lead to an apparent catch‐up response the reason for this is the effect of size on the growth rate of individuals. Yet, measuring the growth at later ages while accounting for individuals’ growth at a previous age enables us to investigate individuals' overall growth pattern more precisely. Therefore, there is a clear need for empirical studies able to tease apart genetic and plastic effects using both growth and growth compensation, based on realistic environmental variables, and using both directional and non‐directional selection processes.

In this study, we focus on the European anchovy Engraulis encrasicolus and the European sardine Sardina pilchardus of the Bay of Biscay. The size‐at age of these two small pelagic fish populations declined substantially during the last two decades but the mechanisms underlying such declines are still largely unknown (Doray et al., 2018; Véron et al., 2020). We have shown previously that there was strong consistent directional selection acting against individuals' with high growth rate at age‐0 in both species but this directional selection is not perfect and it leads to the appearance of a non‐directional (diversifying) selection (Boëns et al., 2021). Directional selection on growth at age‐0 was particularly strong in anchovy when this stock's harvest rate was high (Boëns et al., 2021). The magnitude of selection acting on growth compensation was more limited with signs of diversifying selection again probably due to the imperfect directional selection (Boëns et al., 2021). In anchovies, growth at age‐0 and growth compensation were primarily influenced by biomass (i.e. proxy of density‐dependence), chlorophyll‐a (i.e. proxy of the amount of food) and harvest rate, while in sardines growth at age‐0 only was associated with biomass (Boëns et al., 2021). Our previous study focused on identifying the factors determining temporal changes between cohorts in these two growth parameters and the magnitude of directional and non‐directional selection within cohorts. It is therefore now important to compare the phenotypic characteristics of parental and offspring cohorts to determine whether the selection pressures (directional and non‐directional selection) exerted within parental cohorts have led to an adaptation leading to smaller body size. To this end, we will use otolith growth data collected during fisheries research surveys conducted annually from 2000 to 2018 to parameterize the breeder's equation following the method described in Swain et al. (2007). More specifically, we will quantify the differences in mean and variance in growth at age‐0 and growth compensation at age‐1 between parental cohorts and their offspring (i.e. measuring the responses to directional (mean) and non‐directional (variance) selections). We will then relate these differences to the magnitude of directional and non‐directional selection within parental cohorts while taking into account differences between generations in environmental factors that we previously identified as being the most important for growth and growth compensation (Boëns et al., 2021). If responses to selection are related to selection differentials, the phenotypic changes likely reflect an evolutionary change; if responses to selection are related to differences in environmental factors, phenotypic plasticity likely underpins the phenotypic changes.

## 2 MATERIALS AND METHODS

### 2.1 Sampling at sea and otolith growth measurements

Samples of sardines and anchovies were collected onboard R/V Thalassa during the survey PELGAS (full description in Doray et al., 2018). The survey's spatial coverage comprises the French part of the shelf of the Bay of Biscay, from coast to shelf‐break, and this encompasses most of sardine and anchovy populations in the Bay of Biscay (Doray et al., 2018; Gatti et al., 2017). PELGAS is primarily an acoustic survey that takes place every year in May since 2000 and pelagic trawl hauls are carried out when necessary to identify the species responsible of the echotraces and collect biological parameters for focal species (Doray et al., 2014; Petitgas et al., 2003). After each trawl haul, the catch is sorted and weighed by species and a random subsample is drawn to establish length frequencies and age‐length keys (Doray et al., 2014, 2018). When anchovies and sardines are captured ca. 40 individuals of each species, representative of the established length distribution, are selected to obtain individual measurements. Otoliths are extracted at this stage for age and growth estimation. This is the standard yearly protocol is implemented within the data collection framework for the assessment of fisheries resources managed at EU level (Doray et al., 2014). For this study, the data span 19 years from 2000 to 2018 and comprise 535 and 549 hauls for anchovy and sardine respectively. In the laboratory, we imaged and analysed the otoliths mounted in leukit, with the image processing software TNPC (Mahé et al., 2009). We measured annual growth from segment lengths at age along the longitudinal axis of the otolith between the winter stripes (Boëns et al., 2021). Age‐specific individual growth was measured in all hauls containing anchovy (*N* = 20,185 individuals) and in a selection of stations only for sardine, selected to cover the entire area in each year (*N* = 8264 individuals; details in Boëns et al., 2021).

### 2.2 Parameters measured

Over 90% of individuals' overall growth in anchovy and sardine occurs during the ages 0 and 1 (Petitgas et al., 2012; Uriarte et al., 2016). We, therefore, focused on growth during these key ages (Boëns et al., 2021); the segment R1 up to the first winter ring represented growth at age‐0 and was estimated on individuals of age‐1 and the segment R2 up to the second winter ring represented growth at ages 0 and 1 and was estimated on individuals aged 2. As selection is exerted on fish length rather than on otolith size, we calculated selection differentials based on back‐calculated fish length. To this end, we used the Scale Proportional Hypothesis model (Francis, 1990; Whitney & Carlander, 1956) from the r‐package ‘FSA’ (Ogle et al., 2020). To scale up the individual back‐calculated lengths‐at‐age to anchovy and sardine populations, we calculated their weighted averages following the method described in Boëns et al. (2021) in which the weights represent the proportion of the species's population biomass in the vicinity of the trawl hauls. Here we considered the following growth traits: growth at age‐0, (‘L', estimated from R1) and growth compensation at age‐1 (‘Gc’, estimated from (L2‐L1)/L1: Boëns et al., 2021). Growth compensation represents the ability of a fish with a relatively slow growth rate during the first year (from age 0 to age 1) to undergo more rapid growth during the second year (from age 1 to age 2) (Ali et al., 2003; Metcalfe & Monaghan, 2001; Wright et al., 2007). In our analyses, this parameter, estimated as individuals’ growth at age‐1 (L2‐L1) controlled for their growth at age‐0 (L1) enables us to investigate individuals' growth pattern as (focusing solely on growth at age‐0 would provide only a partial answer to individuals' overall changes in growth).

As explanatory variables, we considered proxies of environmental condition and density‐dependence. We used sea surface temperature and chlorophyll‐a as environmental variables, the latter representing the amount of phytoplankton biomass and by extention the amount of food available in the Bay of Biscay. Data of sea surface chlorophyll‐a and temperature were obtained from the project MARC/Previmer (http://marc.ifremer.fr/) which offers daily satellite images. These were averaged over the French shelf of the Bay of Biscay by quarter in each year (Boëns et al., 2021) because we expected that environmental conditions at particular seasons could influence individuals' growth and/or selective mortality (see Boëns et al., 2021). Yet, as these seasonal variables were strongly related, we carried out a principal components analysis to extract composite variables characterizing the environmental conditions of the Bay of Biscay (details in Boëns et al., 2021). This approach enabled us to limit the risks of over‐parameterization (we only have 18 cohorts) and account for the strong correlation among temperature and chlorophyll‐a data across seasons. The main factor affecting fish growth and the selection processes was the first principal component (‘PC1’ in Boëns et al., 2021), which was primarily related to chlorophyll‐a. Here we used the time series of this component from 1999 to 2020 and explained 39% of the variance in the variables loaded in the PCA (Boëns et al., 2021). Since food is shared among congeners, we also considered population biomass as an explicative variable as individuals' growth can be influenced by density‐dependent mechanisms (e.g. Boëns et al., 2021; Post et al., 1999). To estimate such a density‐dependent effect, we used yearly estimates of anchovy and sardine population abundance as published by ICES (2019) for the Bay of Biscay.

### 2.3 Breeder's equations

We followed the approach of Swain et al. (2007) to estimate the response to directional selection on growth at age‐0 (L) and growth compensation at age‐1 (Gc) in anchovy and sardine:

(1) $$\mathrm{\Delta M}=h^{2}\bar{S}+\mu \Delta E$$

where, ΔM is the mean difference between parents and offspring in the trait considered (either *L* or Gc), $h^{2}$ is the heritability of the trait, $\bar{S}$ is the directional selection within parental cohorts and ΔE is the difference in environmental factors between parents and offspring.

The responses to non‐directional selection on growth at age‐0 (*L*) and growth compensation at age‐1 (Gc) was estimated as:

(2) $$\Delta V=\frac{h^{4}}{2}\left(\bar{C}-{\bar{S}}^{2}\right)+\mu \Delta E^{2}$$

where, ΔV is the difference in the variance of the trait considered (either *L* or Gc) between parents and offspring, $h^{4}$ is the squared trait heritability, $\bar{C}$ is the non‐directional selection within parental cohorts, $\bar{S}$ is the directional selection within parental cohorts and $\Delta E^{2}$ is the difference in squared environmental factors between parents and offspring.

The indices ΔM and ΔV were estimated for the two traits, growth at age‐0 (*L*) and growth compensation at age‐1 (Gc). Equations 1 and 2 are adaptations of the classic breeder's equations (Heywood, 2005; Kelly & Williamson, 2000; Swain et al., 2007). Estimations of each term are described in the following sections.

### 2.4 Estimation of the response indices to selection

The response to directional selection ΔM refers to the change in the mean of growth at age‐0 (*L*) or growth compensation (Gc) over a complete generation. We estimated it as:

(3) $$\Delta M_{j}=M_{j}-\sum_{i=\min\left(\mathrm{age}\right)}^{\max\left(\mathrm{age}\right)}\left(p_{i,j-i}M_{j-i}\right)\;$$

where, *j* is the index for cohorts and *i* for age, $M_{j}$ is the mean of the trait considered (either *L* or Gc) for offspring in cohort *j*, $M_{j-i}$ is the mean of this trait (either *L* or Gc) for parental cohort *j*‐*i*, $p_{i,j-i}$ is the proportion of individuals aged i for the parental cohort *j*‐*i* in the population of parents. For growth at age‐0, three generations were considered in the spawning stock in each year for anchovy (ages 1–3) and four generations were considered for sardine (ages 1–4). For growth compensation, two generations were considered for anchovy (ages 2&3) and three generations for sardine (ages 2–4). Indeed, Bay of Biscay anchovies and sardines are mature at age‐1 and individuals aged greater than 3 and 4 years are rare in these populations (ICES, 2010) respectively. All proportions were calculated to sum to unity.

The response to non‐directional selection ΔV refers to the change in the variance of growth or growth compensation. It is estimated as follows:

(4) $$\Delta V_{j}=V_{j}-\sum_{i=\min\left(\mathrm{age}\right)}^{\max\left(\mathrm{age}\right)}\left(p_{i,j-i}V_{j-i}\right)\;$$

where, *j* is the index for cohorts and *i* for age, $V_{j}$ is the variance of the trait considered (either *L* or Gc) for offpsring in cohort j, $V_{j-i}$ is the variance of this trait (either *L* or Gc) for parental cohort *j*‐*i*, $p_{i,j-i}$ is the proportion of individuals aged i for the parental cohort j‐i in the spawning stock. Consistent with ΔM, all proportions were adjusted to sum to unity. For growth at age‐0, three generations were considered for anchovy and four for sardine while for growth compensation, two generations were considered for anchovy and three for sardine.

We estimated yearly confidence intervals on the response to selection by bootstrapping the individual data following the data structure (Efron, 1992). In each station over the time series, n individuals were randomly sampled with replacement out of the n available individuals (*n* = 40) thus generating a new dataset with which the indices ΔM and ΔV were calculated. The procedure was repeated until convergence of the difference between boostraped and original estimates. We used 250 boostrap repetitions and deduced 95% confidence intervals.

### 2.5 Estimation of the selective mortality and environmental indices

Positive and negative selective mortality differentials applying on trait M reflect the disappearance within the parental cohorts of individuals with small and large values respectively.

These directional selection differentials within the parents of the offspring applying on trait M were estimated as:

(5) $$\bar{S_{j}}=\sum_{i=a}^{\max\left(\mathrm{age}\right)}p_{i,j-i}\left(M_{i,j-i}-M_{a,j-i}\right)\;$$

where, *i* is the index of ages, *j* the index of cohorts, $M_{i,j-i}$ the mean of the trait considered (either *L* or Gc) in the parental cohort *j*‐*i*, $M_{a,j-i}$ the mean of the trait considered at age a for parental cohort *j*‐*i* (for *L*: *a* = 1; for Gc: *a* = 2), and $p_{i,j-i}$ the proportion of individuals aged *i* of the parental cohort *j*‐*i* in the spawning stock in year *j*.

The non‐directional selection differentials within the parents of the offspring were estimated as:

(6) $$\bar{C_{j}}=\sum_{i=a}^{\max\left(\mathrm{age}\right)}p_{i,j-i}\left(V_{i,j-i}-V_{a,j-i}+{\bar{S_{i,j}}}^{2}\right)\;$$

where, *i* is the index of ages, *j* the index of cohorts, $V_{i,j-i}$ the variance of the trait considered (either *L* or Gc) at age i for parental cohort *j*‐*i*, $V_{a,j-i}$ the variance of the trait considered at age a for parental cohort *j*‐*i* (for *L*: *a* = 1; for Gc: *a* = 2), ${\bar{S_{i,j}}}^{2}$ is the directional selection within the parents of the offspring and $p_{i,j-i}$ the proportion of individuals aged *i* for the parental cohort *j*‐*i* in the spawning stock in year *j*.

Finally, ΔE is the difference in the environmental variable E experienced by the offspring cohort and their parents. These differences were estimated as:

(7) $$\Delta E_{j}=E_{j}-\sum_{i=\min\left(\mathrm{age}\right)}^{\max\left(\mathrm{age}\right)}\left(p_{i,j-i}E_{j-i}\right)\;$$

where, *i* is the index of ages, *j* the index of cohorts, $\Delta E_{j}$ the difference in E (Biomass or PC1) between offspring cohort j and their parents, $E_{j-i}$ the Biomass or PC1 for parental cohort *j*‐*i*, $p_{i,j-i}$ the proportion of parental cohort *j*‐*i* (individuals aged i) in the spawning stock in year *j* and $E_{j}$ the mean of the environmental factor considered for the offspring in cohort *j*. Again, all proportions were adjusted to sum to unity. When the environmental parameters were related to growth, three generations were considered for anchovy and four generations for sardine. For growth compensation, two generations were considered for anchovy and three for sardine.

(8) $$\Delta E_{j}^{2}=E_{j}^{2}-\sum_{i=\min\left(\mathrm{age}\right)}^{\max\left(\mathrm{age}\right)}\left(p_{i,j-i}E_{j-i}^{2}\right)\;$$

where, *i* is the index of ages, *j* the index of cohorts, $\Delta E_{j}^{2}$ the difference in $E^{2}$ (Biomass or PC1 squared) between offspring cohort *j* and their parents, $E_{j-i}^{2}$ the Biomass or PC1 squared for parental cohort *j*‐*i*, $p_{i,j-i}$ the proportion of parental cohort *j*‐*i* (individuals aged *i*) in the spawning stock in year j and $E_{j}^{2}$ the mean of the squared environmental factor considered for the offspring in cohort *j*. As for $\Delta E_{j}$, all proportions were adjusted to sum to unity. For growth, three generations were considered for anchovy and four for sardines. For growth compensation, two generations were considered for anchovy and three for sardine.

### 2.6 Statistical analysis

**FIGURE A1:**
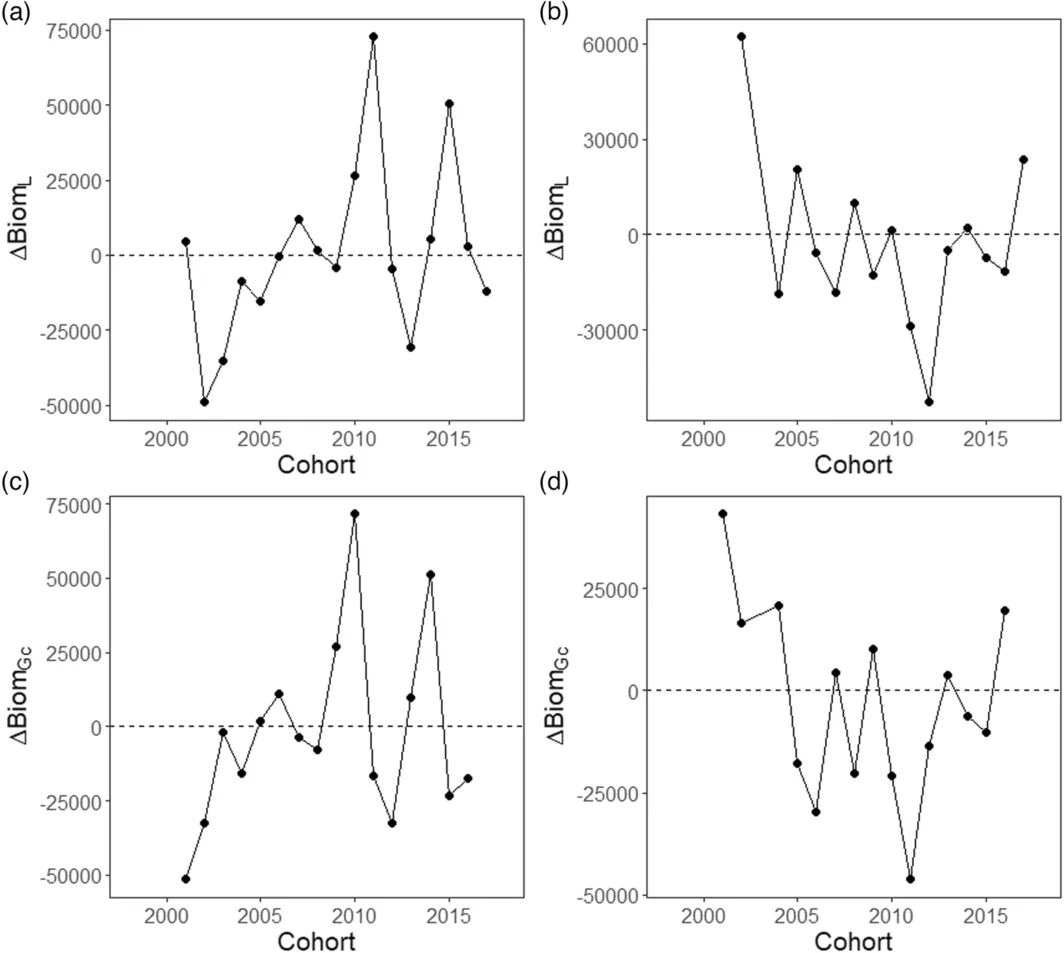
Temporal changes in the difference in biomass linked to growth at age‐0 ($\text{ΔBio}m_{L}$; Figure a, b) and in the difference in biomass linked to growth compensation ($\text{ΔBio}m_{\mathrm{Gc}}$; Figure c and d) for anchovies (a, c) and sardines (b, d).

**FIGURE A2:**
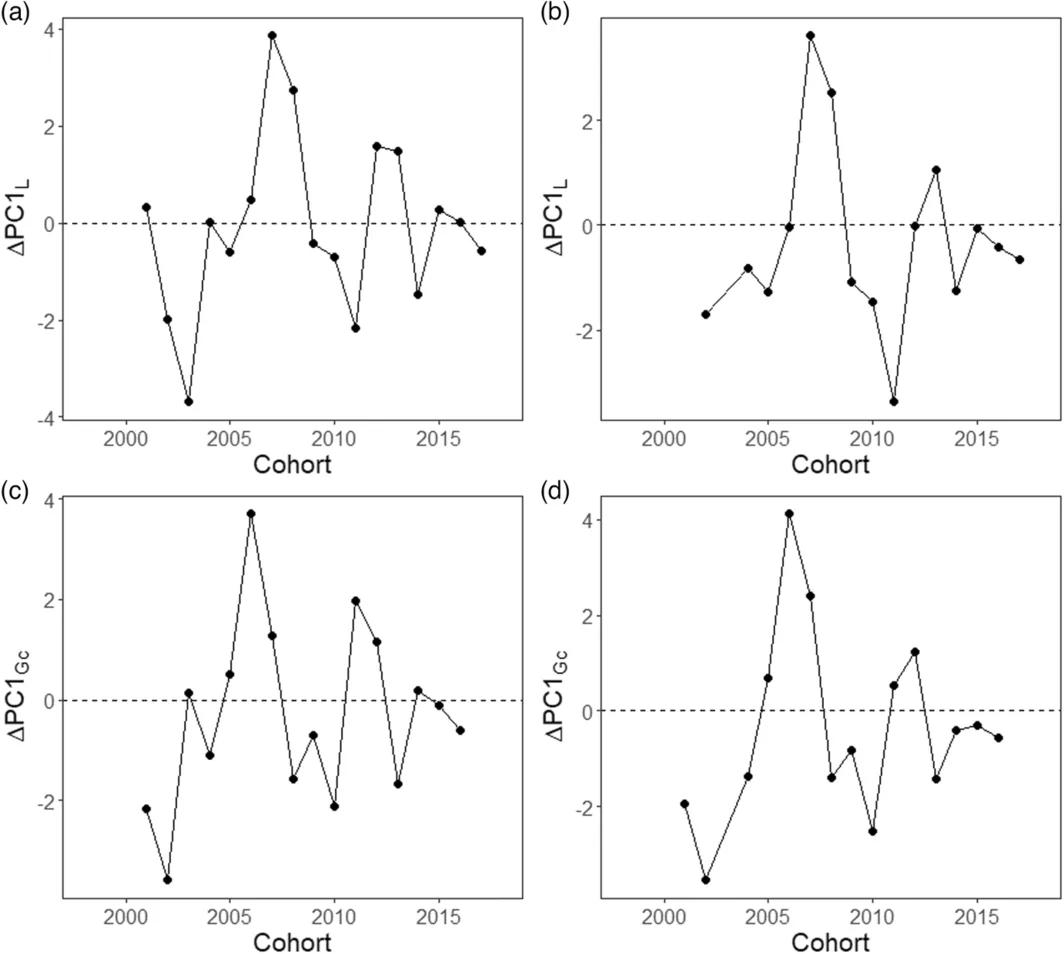
Temporal changes in the difference in chlorophyll‐a linked to growth at age‐0 ($\mathrm{\Delta PC}1_{L}$; Figure a, b) and in the difference in chlorophyll‐a linked to growth compensation ($\mathrm{\Delta PC}1_{\mathrm{Gc}}$; Figure c, d) for anchovies (a, c) and sardines (b, d). PC1 refers to the first principal component summarizing environmental parameters, chlorophyll‐a being highly correlated with that component.

**FIGURE A3:**
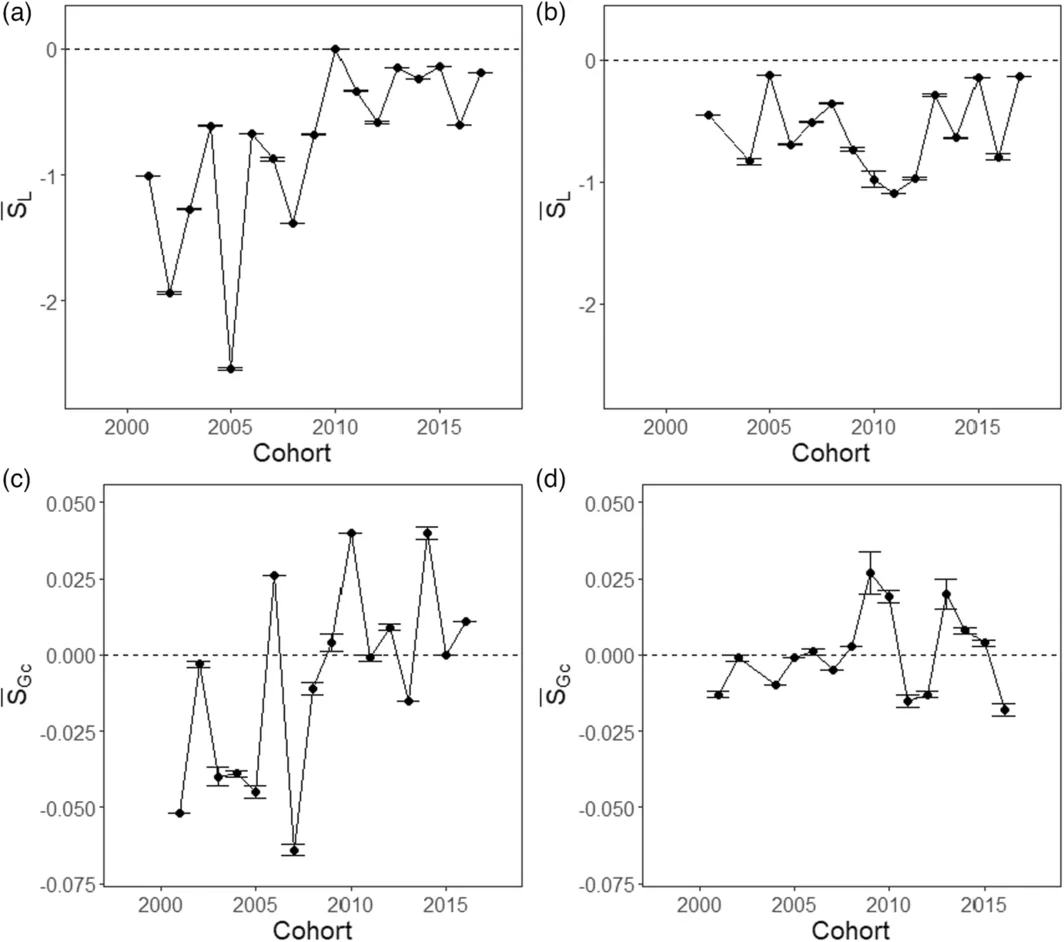
Temporal changes in directional selection linked to growth at age‐0 (${\bar{S}}_{L}$; Figure a, b) and in that linked to growth compensation at age‐1 (${\bar{S}}_{\mathrm{Gc}}$; Figure C, D) for anchovies (a, c) and sardines (b, d).

**FIGURE A4:**
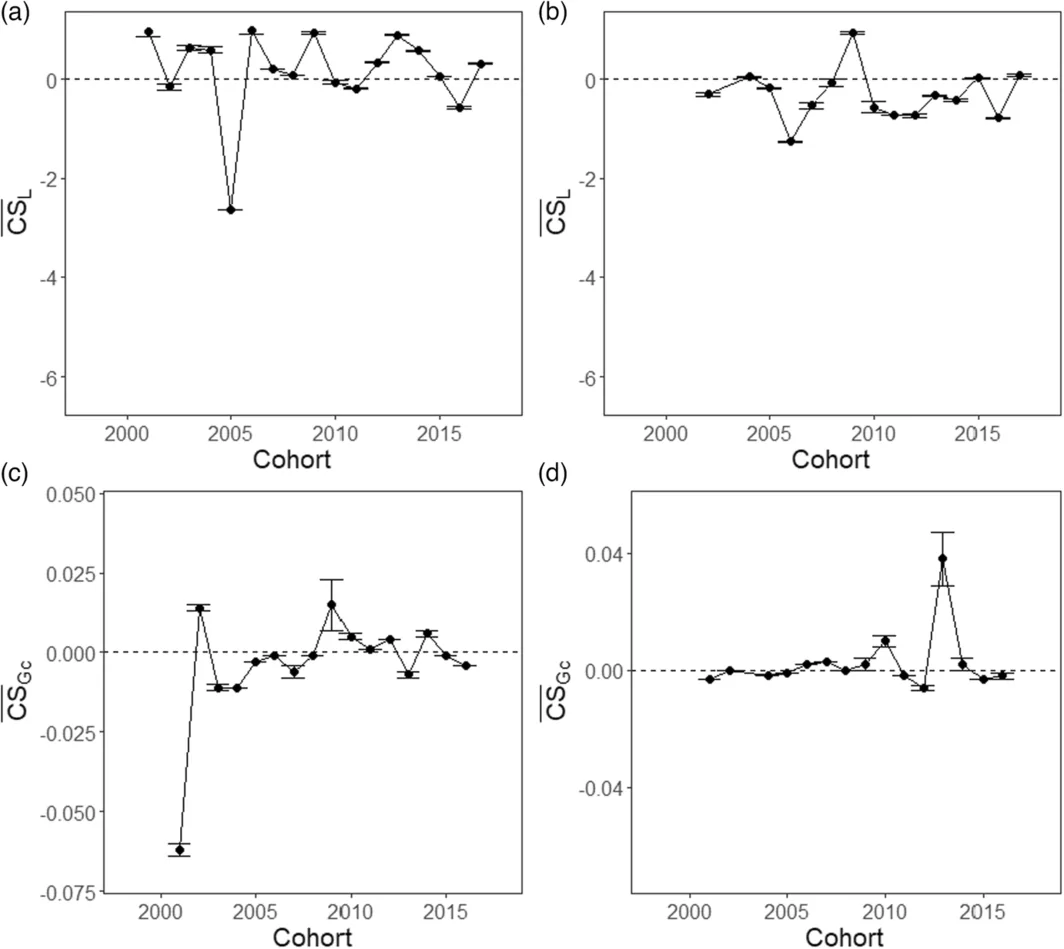
Temporal changes in non‐directional selection linked to growth at age‐0 (${\bar{\mathrm{CS}}}_{L}$; Figure a, b) and in non‐directional selection linked to growth compensation (${\bar{\mathrm{CS}}}_{\mathrm{Gc}}$; Figure c, d) for anchovies (a, c) and sardines (b, d). ${\bar{\mathrm{CS}}}_{L}$ = ${\bar{C}}_{L}$ – ${\bar{S}}_{L}^{2}$ and ${\bar{\mathrm{CS}}}_{\mathrm{Gc}}$ = ${\bar{C}}_{\mathrm{Gc}}$ – ${\bar{S}}_{\mathrm{Gc}}^{2}$ (see Equation 2, Table 3).

Equations 1 and 2 were fitted using linear models. Dependent and explanatory variables were all centred and normed prior to model fitting (i.e. for each parameter, values were subtracted by the average and divided by the standard deviation). Full models contained the responses to selection ($\Delta M_{L}$ or $\Delta M_{\mathrm{Gc}}$ for Equation 1; $\Delta V_{L}$ or $\Delta V_{\mathrm{Gc}}$ for Equation 2) and all the explanatory variables ($\bar{S}$, ΔBiom and ΔPC1 for Equation 1; the difference $\bar{C}$‐${\bar{S}}^{2}$, $\text{ΔBio}m^{2}$ and $\mathrm{\Delta PC}1^{2}$ for Equation 2; Figures A1, A2, A3, A4). These full models were subsequently simplified using the ‘dredge’ function and models were ranked according to Akaike's Information Criterion (AIC) using the r‐package ‘MuMIn’ v1.43.17 (Barton, 2017). Model‐averaged coefficient estimates with unconditional SE and unconditional 95% CI were subsequently calculated for models with differences in corrected AIC (AICc) lower than 2. Note that the use of linear models to estimate the parameters of the breeder's equation entails the estimation of a constant (the intercept) which captures the variance in the response to selection not explained by the parameters included in the models. This unexplained change in *L* or Gc can either be due to other environmental variables or episodes of selection not estimated by ${\bar{S}}_{L}$, ${\bar{S}}_{\mathrm{Gc}}$, ${\bar{C}}_{L}$, or ${\bar{C}}_{\mathrm{Gc}}$ (Heino et al., 2008). As the process of model selection and parameter averaging can lead to large estimates of standard errors (i.e. due to both the uncertainties in parameter estimates but also in model selection, Anderson & Burnham, 2002), we tested the robustness of our estimates using Jackknifing (allows to directly estimate both the bias and the variance of the estimation). We created new datasets from the datasets, containing the response to the selection with all the explanatory variables by removing 1 year at a time. We then estimated the average coefficients from models already simulated with the ‘dredge’ function for each new dataset with 1 year less and calculated the mean and standard deviation of the set of mean coefficients calculated with the Jackknife method. All analyses were run in R 4.0.2 (R Core Team, 2014). The list of variables and their abbreviations is available in Table 1.

**TABLE 1:** List of the variables and their abbreviations considered in the modelling.

Abbreviation	Definition
$\Delta M_{L}$	Difference in mean growth at age‐0 between parents and offspring
$\Delta V_{L}$	Difference in variances in growth at age‐0 between parents and offspring
$\Delta M_{\mathrm{Gc}}$	Difference in mean catch up growth at age‐1 between parents and offspring
$\Delta V_{\mathrm{Gc}}$	Difference in variances in catch up growth at age‐1 between parents and offspring
ΔE	Difference in environmental factors between parents and offspring
$\Delta E^{2}$	Difference in squared environmental variables between parents and offspring
$h^{2}$	Trait heritability
${\bar{S}}_{L}$	Mean directional selection within parental cohorts related to growth at age‐0
${\bar{S}}_{\mathrm{Gc}}$	Mean directional selection within parental cohorts related to growth compensation at age‐1
${\bar{C}}_{L}$	Mean non‐directional selection within parental cohorts applied to growth at age‐0
${\bar{C}}_{\mathrm{Gc}}$	Mean non‐directional selection within parental cohorts applied to growth compensation at age‐1
${\bar{\mathrm{CS}}}_{L}$	Difference between ${\bar{C}}_{L}$ and ${\bar{S}}_{L}^{2}$
${\bar{\mathrm{CS}}}_{\mathrm{Gc}}$	Difference between ${\bar{C}}_{\mathrm{Gc}}$ and ${\bar{S}}_{\mathrm{Gc}}^{2}$

## 3 RESULTS

### 3.1 Growth and growth compensation

**FIGURE 1:**
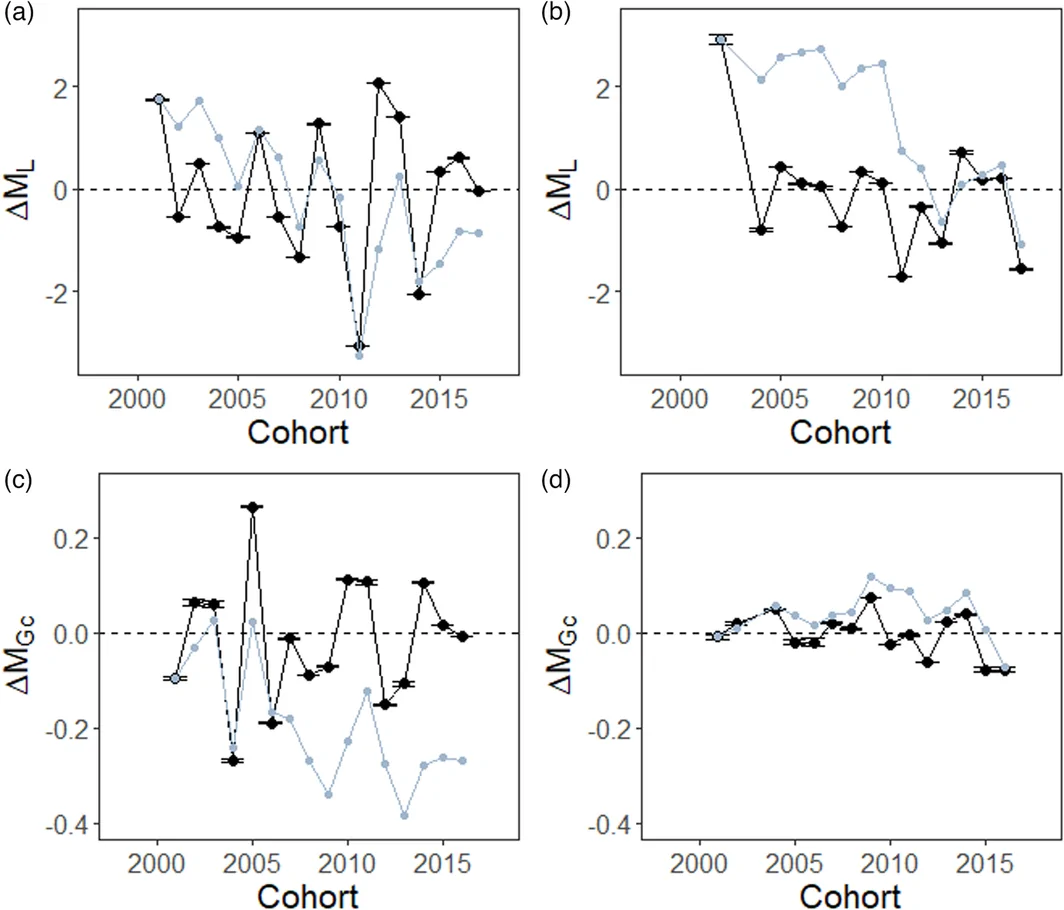
Temporal variations in mean differentials of growth at age‐0 (ΔL; panels a and b) and mean growth compensation at age‐1 (ΔGc; panels c and d) for anchovy (panels a and c) and sardine (panels b and d) with their yearly 95% confidence intervals. The cumulative sum reflects the overall response of the parameter investigated. For each parameter is shown in grey.

From 2001 to 2017, the overall difference in mean growth between parents and offspring ($\Delta M_{L}$) is negative for anchovy and sardine (Cumulative sum $\Delta M_{L}$; anchovy: −0.87; sardine: −1.09, Figure 1a,b). Therefore, despite significant interannual variations, offspring have smaller mean lengths at age‐0 than their parents. In anchovy, this decrease is consistent over time (linear regression: β ± SE = −0.015 ± 0.071; *p* = 0.84, Figure 1a) while in sardine, the decline has been slightly steeper in recent years (linear regression: β ± SE = −0.108 ± 0.060; *p* = 0.10; Figure 1b). The difference in mean growth compensation between parents and offspring ($\Delta M_{\mathrm{Gc}}$) is also mainly negative for anchovies and sardines (Cumulative sum $\Delta M_{\mathrm{Gc}}$; anchovy: −0.27; sardine: −0.07; cohorts 2001–2016, Figure 1C,D). Thus, offspring have, on average, a lower mean growth compensation than their parents. In both species, the difference is consistent through time (linear regression; anchovy: β ± SE = 0.002 ± 0.008; *p* = 0.76, Figure 1C; sardine: β ± SE = −0.004 ± 0.002; *p* = 0.14, Figure 1D). For both $\Delta M_{L}$ and $\Delta M_{\mathrm{Gc}}$, 95% confidence intervals were particularly small and rarely overlapped with zero (Figure 1) and the year‐to‐year variations in anchovy were larger than in sardine.

### 3.2 Variance in growth and growth compensation

**FIGURE 2:**
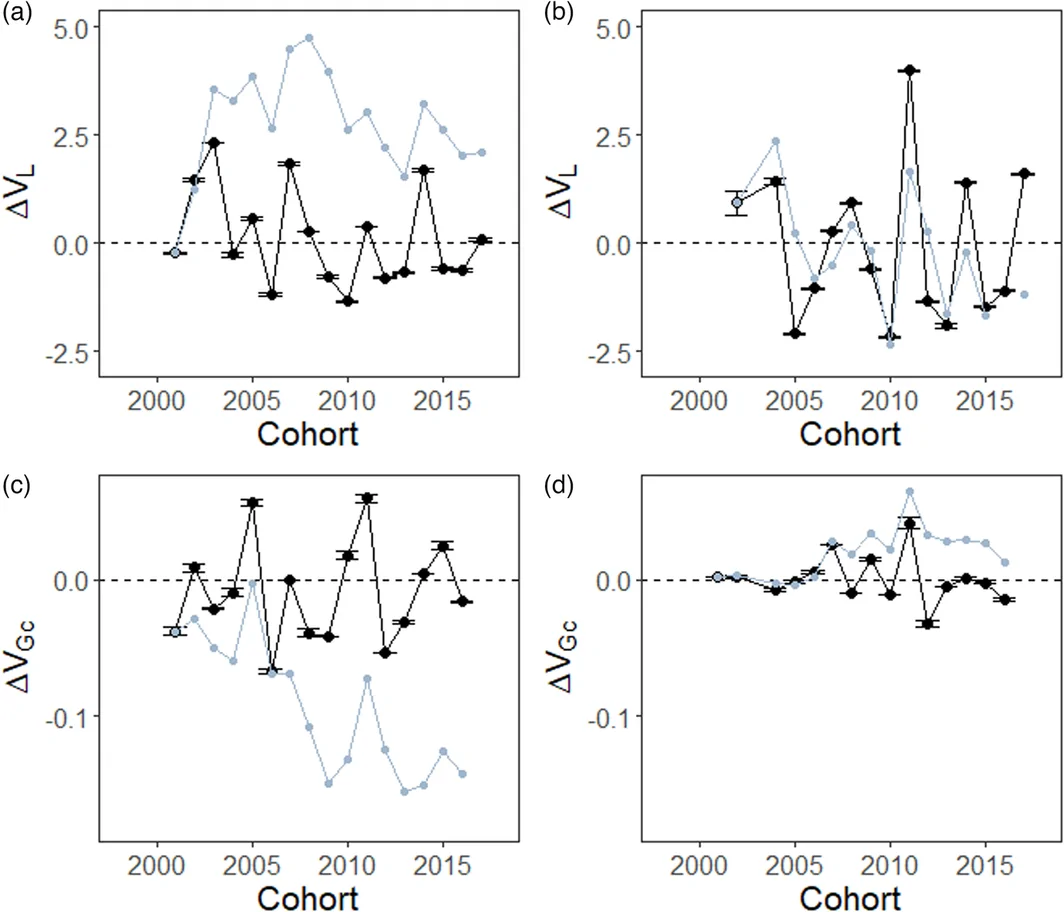
Temporal variation in differentials of variances in growth at age‐0 ($\Delta V_{L}$; panels a and b) and variances in growth compensation at age‐1 ($\Delta V_{\mathrm{Gc}}$; panels c and d) for anchovy (panels a and c) and sardine (panels b and d) with their yearly 95% confidence intervals. The cumulative sum reflects the overall response of the parameter investigated. For each parameter is shown in grey.

The difference in variances in growth at age‐0 between parents and offspring ($\Delta V_{L}$) is positive for anchovy (Cumulative sum $\Delta V_{L}$: 2.098; Figure 2a) and negative for sardines (Cumulative sum $\Delta V_{L}$: −1.186; Figure 2b), on average over the entire time series. Therefore, anchovy and sardine offspring have, respectively, greater and smaller variances in growth at age‐0 than their parents (again with marked differences between years). In both species, there were no temporal patterns in $\Delta V_{L}$ (linear regression; anchovy: β ± SE = −0.069 ± 0.054; *p* = 0.22, Figure 2a; sardine: β ± SE = −0.017 ± 0.106; *p* = 0.88, Figure 2b). The difference in the variance in growth compensation between parents and offspring ($\Delta V_{\mathrm{Gc}}$) is negative for anchovy (Cumulative sum $\Delta V_{\mathrm{Gc}}$: −0.142; Figure 2C), and slightly positive for sardine (Cumulative sum $\Delta V_{\mathrm{Gc}}$: 0.013; Figure 2D). The year‐to‐year variation was greater in anchovy than in sardine. Consistent with $\Delta V_{L}$, there were no temporal patterns in $\Delta V_{\mathrm{Gc}}$ (linear regression; anchovy: β ± SE = 0.001 ± 0.002; *p* = 0.71, Figure 2C; sardine: β ± SE = −0.001 ± 0.001; *p* = 0.53, Figure 2D).

### 3.3 Models fitted using the breeder's equations

**FIGURE 3:**
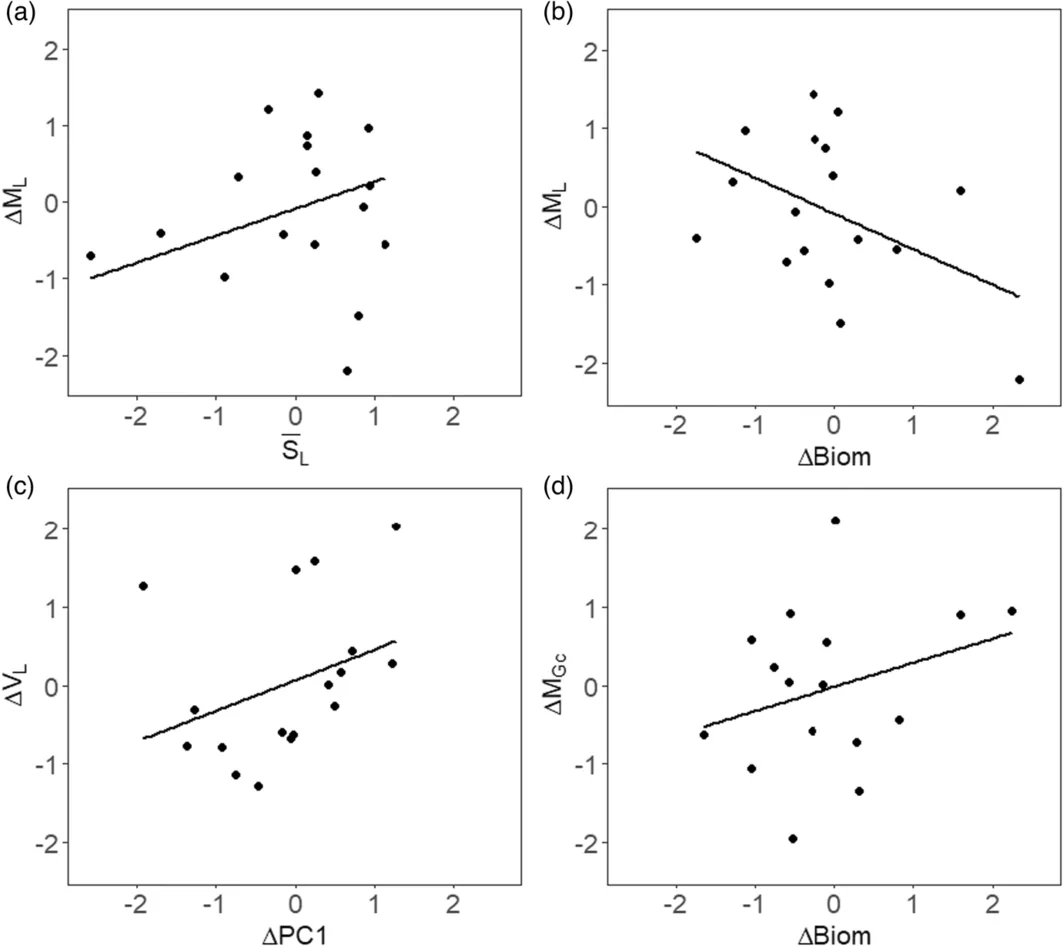
Relationships explaining in anchovy the difference between parents and offspring in the mean and variance in growth at age‐0 ($\Delta M_{L}$, $\Delta V_{L}$), and the mean and variance in growth compensation at age‐1 ($\Delta M_{\mathrm{Gc}}$, $\Delta V_{\mathrm{Gc}}$).

In anchovy, there were five models with ΔAICc < 2 for the difference in mean length between parents and offspring (Table 2a). All explanatory variables of the breeder's equation appeared in these models (i.e. differences in biomass, selection differentials, differences in PC1) but the difference in biomass was present in three of the five models (Table 2a). The averaged parameter estimates showed that $\Delta M_{L}$ was negatively related to ΔBiom, slightly positively related to ΔPC1 (Table 2a; Table A1; Figure 3a,b) and positively related to ${\bar{S}}_{L}$. These parameter estimates and SD were very consistent when Jackknifing the dataset indicating that they are very robust (Table A1). Therefore, offspring growth at age‐0 increased relative to that of their parents when population biomass decreased and the amount of food increased and vice versa. Furthermore, parental generations with high selective mortality produced offspring with lower growth at age‐0, suggesting an adaptive process. The estimated heritability parameter for growth at age‐0 is biologically realistic ($h^{2}$ = 0.35), its 95% confidence interval is relatively wide (−0.10–0.86) but this estimate is very robust (mean $h^{2}$ estimated by Jackknife: 0.35, SD = 0.05). The uncertainty in model selection is probably high generating large confidence intervals for the parameter estimates in the average model. But the Jacknife procedure results in robust estimates with low SD, meaning that similar estimates are obtained although different models are possible (model uncertainty) given the variability in the data series. Large confidence intervals were also found for the other modelled coefficients of ΔBiom and ΔPC1, which reflects the important variations between years, the uncertainty in model selection, and thus relatively weak modelled effects (as evidenced by the presence of the null model in the models with ΔAICc < 2, Table 2a). For the variance in growth at age‐0 ($\Delta V_{L}$), there were two models with ΔAICc < 2 (including the null model). The variance in growth at age‐0 ($\Delta V_{L}$) decreased when the difference in food quantity increased but again confidence intervals of these coefficients were large (Table 2b; Table A2; Figure 3c). For the difference in mean and variance in growth compensation ($\Delta M_{\mathrm{Gc}}$ and $\Delta V_{\mathrm{Gc}}$) the best models were the null models. For $\Delta M_{\mathrm{Gc}}$, there was a small positive effect of ΔBiom (Table 2c; Table A3; Figure 3D) suggesting that an increase in biomass between the parental and offspring cohorts induces a slight increase in mean growth compensation. None of the parameters explained the variation of $\Delta V_{\mathrm{Gc}}$ (Table 2d; Table A4).

**TABLE 2:** Fitted models with ΔAICc < 2 explaining the difference in anchovy growth at age‐0 and growth compensation at age‐1 between parents and offspring. Linear models are fitted using three explanatory variables (ΔBiom: difference in biomasse; $\bar{S}$: mean directional selection; ΔPC1: difference in environmental principal component PC1 reflecting the amount of food available) and ranked by decreasing values of the corrected Akaike's information criterion.

	Dependent variable	Model	logLik	AICc	AICc	Wi	Cum.Wi
a)	Difference in mean growth ($\Delta M_{L}$)	ΔBiom	−21.74	51.33	0.00	0.30	0.30
		Intercept	−23.25	51.36	0.03	0.30	0.60
		ΔBiom + $\bar{S}$	−20.68	52.69	1.36	0.15	0.75
		ΔBiom + ΔPC1	−20.85	53.03	1.70	0.13	0.88
		ΔPC1	−22.69	53.23	1.90	0.12	1.00
b)	Difference in growth variances ($\Delta V_{L}$)	Intercept	−23.75	52.35	0.00	0.60	0.60
		ΔPC1^2^	−22.64	53.13	0.78	0.40	1.00
c)	Difference in mean growth compensation ($\Delta M_{\mathrm{Gc}}$)	Intercept	−22.62	50.17	0.00	0.69	0.69
		ΔBiom	−21.88	51.76	1.59	0.31	1.00
d)	Difference in growth compensation variances ($\Delta V_{\mathrm{Gc}}$)	Intercept	−14.96	34.85	0.00	1.00	1.00

Abbreviations: AICc, corrected Akaike's information; Cum.Wi, cumulative model weight; logLik, log likelihood; Wi, model weight; ΔAICc, difference in AICc values between the current model and that having the lowest AICc.

**FIGURE 4:**
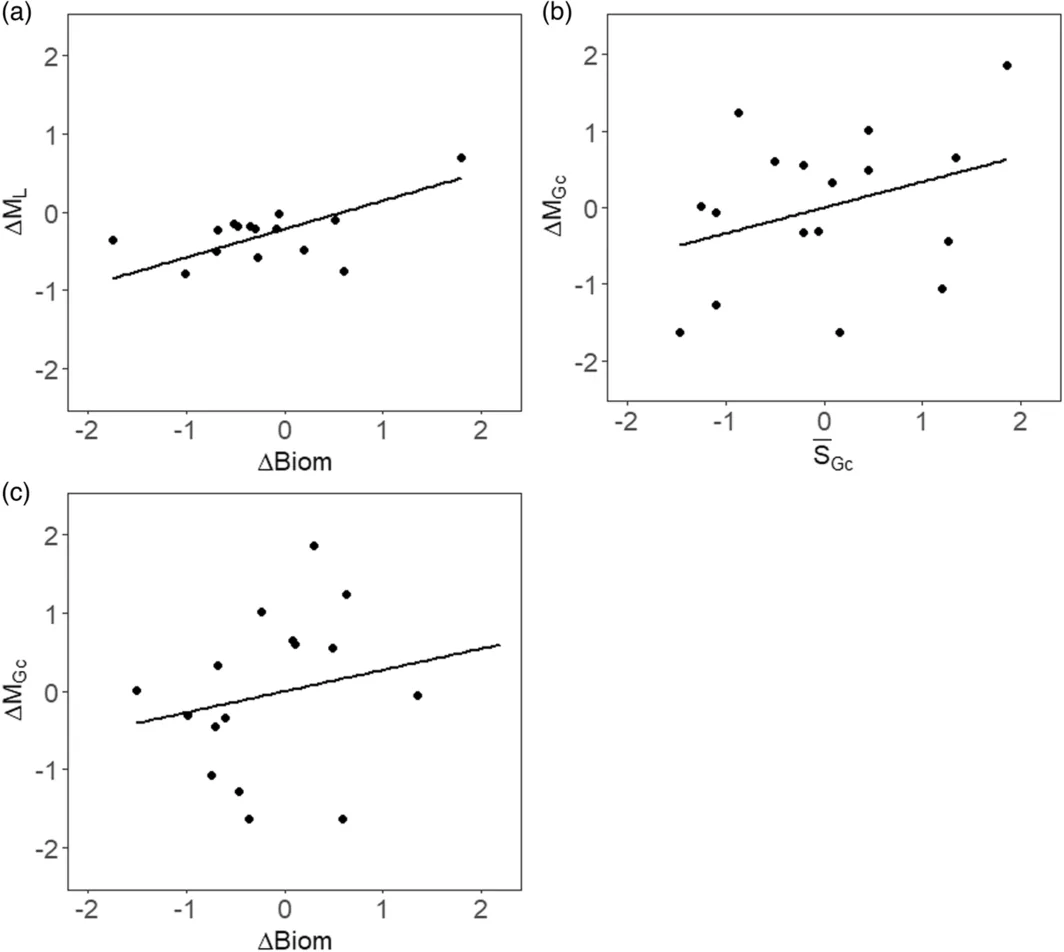
Relationships explaining in sardine the difference between parents and offspring in the mean and variance in growth at age‐0 ($\Delta M_{L}$, $\Delta V_{L}$), and the mean and variance in growth compensation at age‐1 ($\Delta M_{\mathrm{Gc}}$, $\Delta V_{\mathrm{Gc}}$).

In sardine, $\Delta M_{L}$ was strongly and positively related to the difference in biomass between parents and offspring (ΔBiom; Table 3a; Table A5; Figure 4a) with offspring being substantially smaller than their parents when population abundance was lower. None of the explanatory variable explained the variations in $\Delta V_{L}$ (Table 3b; Table A6). For the difference in mean growth compensation ($\Delta M_{\mathrm{Gc}}$) between parents and offspring, three models had ΔAICc < 2 (Table 3c). The null model was the best but both differences in biomass (ΔBiom) and selective mortality (${\bar{S}}_{\mathrm{Gc}}$) explained some of the variance in $\Delta M_{\mathrm{Gc}}$ (Table 3c; Table A7; Figure 4b,C). Consistent with $\Delta V_{L}$, the best model for $\Delta V_{\mathrm{Gc}}$ was the null model thus none of the explanatory variables were retained as explicative (Table 3d; Table A8). Thus, density‐dependent effects explained parent‐offspring differences in mean growth at age‐0 and growth compensation in sardine, offspring having greater growth and catch up growth than their parents when population abundance increased. The genetic effects were clearly less important in this species for the differences in growth at age‐0 since the models comprising ${\bar{S}}_{L}$ had ΔAICc> 2 (Table 3), whereas this parameter appeared in the best models for $\Delta M_{\mathrm{Gc}}$ (Table 3c). The estimated coefficient of ${\bar{S}}_{L}$ reflects the heritability of growth compensation ($h^{2}$ = 0.34), its 95% confidence interval again overlaps with zero (−0.12–0.82) but this parameter estimates varies little following Jackknifing (Table A7).

**TABLE 3:** Fitted models with ΔAICc< 2 explaining the difference in sardine growth at age‐0 and growth compensation at age‐1 between parents and offspring. Linear models are fitted using three explanatory variables (ΔBiom: difference in biomasse; $\bar{S}$: mean directional selection; ΔPC1: difference in environmental principal component PC1 reflecting the amount of food available) and ranked by decreasing values of the corrected Akaike's information criterion.

	Dependent variable	Model	logLik	AICc	ΔAICc	Wi	Cum.Wi
a)	Difference in mean growth ($\Delta M_{L}$)	ΔBiom	−2.46	13.09	0.00	1.00	1.00
b)	Difference in growth variances ($\Delta V_{L}$)	Intercept	−21.63	48.25	0.00	1.00	1.00
c)	Difference in mean growth compensation ($\Delta M_{\mathrm{Gc}}$)	Intercept	−23.61	52.07	0.00	0.49	0.49
		$\bar{S}$	−22.59	53.02	0.95	0.30	0.79
		ΔBiom	−22.96	53.76	1.69	0.21	1.00
d)	Difference in growth compensation variances ($\Delta V_{\mathrm{Gc}}$)	Intercept	−23.61	52.07	0.00	1.00	1.00

Abbreviations: AICc, corrected Akaike's information; Cum.Wi, cumulative model weight; logLik, log likelihood; Wi, model weight; ΔAICc, difference in AICc values between the current model and that having the lowest AICc.

## 4 DISCUSSION

### 4.1 Parents and offspring differences in the mean and variance in growth and growth compensation

Over the entire time series, we observed a decline in growth for both species between generations, whereas for growth compensation, there was a decline for anchovy only and not for sardine (as demonstrated by the cumulative sums). Thus, for anchovy, it is the whole growth process that has changed between generations (growth at age‐0 and growth compensation at age‐1) which explains the strong decline in size‐at‐age observed in this species in the Bay of Biscay (Boëns et al., 2021; Doray et al., 2018). In sardine, while growth at age‐0 strongly decreased, growth compensation at age‐1 did not change. The maintenance of growth compensation in sardine, might explain why its decline in size‐at‐age is less important in their second year of life (Doray et al., 2018; Véron et al., 2020). Such inter‐generational declines have already been observed in exploited fish populations (Atlantic cod: Sinclair et al., 2002; Swain et al., 2007; Northern pike: Edeline et al., 2007; Alpine whitefish: Nusslé et al., 2009), for which fishery‐related evolutionary changes have been demonstrated. These patterns are similar to those observed within cohorts previously (Boëns et al., 2021). Here, inter‐generational analyses allowed us to understand the phenotypic variability generated by anthropogenic and/or environmental factors. These inter‐generational changes may have important consequences for anchovy and sardine stocks in the Bay of Biscay as the largest fish within each cohort in marine fish are often those with higher survival rates and reproductive investments (Gjerde, 1986; Johns et al., 2018; Riebe et al., 2014).

In anchovy, the variance in growth at age‐0 overall increased between parents and offspring, resulting in offspring having a higher frequency of low‐ and high‐growth individuals at age‐0 than their parents. There are as many years with positive (7 years) and negative (9 years) differences, but the overall increase in variance is mainly due to a few years (2002, 2003, 2007 and 2014) for which the variance in growth at age‐0 of the offspring was much higher than that of their parents. This increase in variance in particular years may be explained by three non‐exclusive processes: (i) a low variance in parental growth, (ii) particularly favourable environmental conditions (temperature, food, low predation) allowing the increase in growth of some individuals and the survival of those with slow growth and (iii) significant heterogeneity in environmental conditions leading to an increase in growth differences between individuals having lived in favourable and unfavourable areas. As there was no significant difference between the variance of the parental generations, it is likely that this increase in variance in the offspring for particular years was due to better environmental conditions for the offspring. Indeed, the spatial structuring of the pelagic environment in the Bay of Biscay can be very variable (e.g. Petitgas et al., 2018). In sardines, the variance in growth at age‐0 was generally lower in offspring than in parents (Figure 2b) and the offspring generations had generally fewer individuals with high and low growth values at age‐0 than their parents. Such declines in variance in growth within and between generations are relatively common and have been reported in aquatic environments for species as varied as Antlantic Cod (Olsen et al., 2009; Sinclair et al., 2002; Swain et al., 2007) but also in terrestrial environments (Hamel et al., 2016). While the decline in within cohort variance in growth is usually attributed to catch up growth and selective mortality acting on individuals with fast or slow growth, the among generation decline in variance in growth was probably associated with the declines in average growth and the constraints set by environmental factors on individuals' growth.

### 4.2 Genetic effects on growth and growth compensation

Determining whether evolutionary processes are involved in the changes in the phenotypic characteristics of natural populations is important because adaptive changes may influence their long‐term dynamics by modifying their productivity (Dunlop et al., 2015; Jørgensen et al., 2007; Laugen et al., 2014) and may cause changes in traits reversible only over several generations (Dieckmann & Heino, 2007; Enberg et al., 2009; Jørgensen et al., 2007; Law, 2000). In our case, directional selection was always less than zero for anchovy and sardine, meaning that there is a disappearance of high‐growth individuals at age‐0 within parental cohorts (Boëns et al., 2021). We have already shown that this effect was explained by fishing pressure in anchovy and that none of the variables we used explained the variance in this selective mortality in sardine (Boëns et al., 2021). In anchovy, we found a positive effect of ${\bar{S}}_{L}$ on $\Delta M_{L}$, which indicates that the stronger the selective disappearance of large individuals within parental generations, the lower the growth of their offspring compared to their parents. This effect is particularly strong during the first part of the time series and then diminishes, as ${\bar{S}}_{L}$ is almost no longer variable while $\Delta M_{L}$ is still very variable (Figure 3a). These two processes (at the beginning and at the end of the series) may explain why the overall variability explained is relatively low and the wide confidence interval observed. The effect of ${\bar{S}}_{L}$ on $\Delta M_{L}$ in anchovy suggests that directional selection, particularly strong at the beginning of the time series when fishing effort was high, induced an adaptive response leading to a decline in growth at age‐0 before and during the moratorium. As 80% of the total growth of anchovy is due to growth at age‐0, this adaptation largely explains the overall decline in size‐at‐age of this species during early 2000s. Following the moratorium, the stock biomass recovered and fishing pressure remained moderate but anchovy growth remained low (Boëns et al., 2021). A density‐dependent process probably took place that limited individuals' growth, which explains the variations in $\Delta M_{L}$ without any link with ${\bar{S}}_{L}$ (see Figure 3a) and also limited the effect of natural selection that could have favoured individuals with high‐growth rate at age‐0 once the harvest rate declined. Remarkably, the selection regime has changed in anchovy for ${\bar{S}}_{\mathrm{Gc}}$ with very strongly negative values at the beginning of the time series and positive values at the end of the time series (Figure A3). Therefore, the weak overall effect of selective mortality may be due to a double response: first, an adaptive response leading to a decrease in growth at age‐0 and in which fish with high growth compensation at age‐1 had high mortality rates (before the moratorium) followed by a period in which individuals with higher growth compensation survived longer (Boëns et al., 2021). This result indicates that it is the overall growth pattern of anchovy which has changed in response to the strong harvest rate in the early 2000s. All large fish (i.e. those with large growth at age‐0 and those with low growth at age‐0 but high growth compensation at age‐1) rapidly disappeared within cohorts. Thus, the substantial decline in anchovy growth due to some extent to fishing in the first half of the 2000s may have contributed to this stock's collapse through both a decline in biomass and a strong changes in individuals' growth pattern.

In sardine, we found no effect of ${\bar{S}}_{L}$ on $\Delta M_{L}$ but ${\bar{S}}_{\mathrm{Gc}}$ had a positive effect on $\Delta M_{\mathrm{Gc}}$. As the time series of ${\bar{S}}_{\mathrm{Gc}}$ (Figure A3) has both negative and positive values, selective mortality favours individuals with strong growth compensation in some years but disadvantaging them in others. Most ${\bar{S}}_{\mathrm{Gc}}$ are close to zero and few cohorts show strong positive ${\bar{S}}_{\mathrm{Gc}}$. Therefore, parental cohorts in which fish with higher growth compensation at age‐1 survived longer produced offspring with greater (on average) growth compensation (processes unrelated to fishing: Boëns et al., 2021). This adaptive growth compensation response probably may have resulted in the maintenance of growth compensation for this species and thus a smaller decline in size‐at‐age 2 for sardine in comparison to anchovy (we did not find an adaptive response of growth compensation in anchovy probably because of a weaker relationship between harvest rate and this trait, which is probably also strongly linked to environmental factors). We found no effect of non‐directional selection on the means and variances of L or Gc. This may be due to the generally weaker effects of non‐directional selection compared with directional selection (Kingsolver et al., 2001), but in our case, the apparent diversifying selection was also probably mainly due to the imperfect directional selection (the increase in variance was primarily due to a decline in the frequency of the most common phenotypes associated with the survival of some individuals with large growth at age‐0; Boëns et al., 2021). Therefore, it is not very surprising that the consequences of this apparent diversifying selection on the phenotypic characteristics of the offspring were limited. Based on our analyses, we estimated the heritability of growth at age‐0 in anchovy to 0.35 and the heritability of sardine growth compensation to 0.34. These values are consistent with those previously estimated in nature (Law, 2000; Nusslé et al., 2009; Swain et al., 2007) and in laboratory (Garcia de Leaniz et al., 2007).

### 4.3 Effects of environmental factors on the response to selection

The growth of organisms is strongly influenced by multiple environmental factors that underpin their phenotypic plasticity (e.g. Baudron et al., 2014; Brosset et al., 2016; Daufresne et al., 2009; Ohlberger, 2013). In anchovy, there is a negative effect of ΔBiom on $\Delta M_{L}$ and $\Delta V_{L}$, meaning that the offspring living in a population whose biomass is greater than that of their parents have a lower mean and variance of growth at age‐0. This density‐dependence effect on growth has already been observed in pelagic fish (e.g. Brunel & Dickey‐Collas, 2010) and is implicit in many studies investigating density‐dependence even though measures used are cohort‐level growth measures rather than parent‐offspring differences. The mechanism underlying the density‐dependence of growth in anchovy could be based on the increase in competition for food between individuals with the increase in biomass between parents and offspring (e.g. Enberg et al., 2009; Lorenzen & Enberg, 2002; Schram et al., 2006; Svedäng & Hornborg, 2014). We also found a positive effect of ΔPC1 on $\Delta V_{L}$ in anchovy, meaning that when the environment of the offspring shows greater chlorophyll‐a concentrations (proxy for food) than that of their parents, the variability in the growth at age‐0 in the offspring is larger. Differences in growth compensation between parents and offspring were only related to the differences in biomass between generations, therefore offspring have a higher growth compensation compared to their parents when they live in a higher biomass population. This relationship is surprising because it is opposite to the one linking differences in biomass with differences in growth at age‐0, but can be explained by the negative correlation between growth at age‐0 and growth compensation at age‐1 (Boëns et al., 2021). Hence, fish with limited growth at age‐0 invest more energy in growth subsequently (at age‐1), while fish with high growth rate at age‐0 can invest more energy in their reproductive effort at age‐1. This result is consistent with the size structure of this stock, as fish with high growth rate at age‐0 live offshore at age‐1 in the area where egg densities are large. None of the explanatory variables were related to $\Delta V_{\mathrm{Gc}}$ and it is possible that other variables not used here could also contribute to explain the variance in this parameter.

In sardine, differences in average growth at age‐0 and growth compensation at age‐1 are only linked to ΔBiom. Therefore, unlike in anchovy, the sardine offspring living in environments with a greater biomass (strongly related to their abundance) than their parents have an overall growth that increased. This relationship can be explained by a bottom‐up process in which individuals' growth and populations' biomass increase under favourable conditions and if sardines' range expands to limit intraspecific competition. None of the explanatory variables we used could explain $\Delta V_{L}$ and $\Delta V_{\mathrm{Gc}}$. Growth is strongly linked to environmental variations (such as temperature, food quantity or quality) making it unsurprising to find such large effects of phenotypic plasticity in our two species.

### 4.4 Limitations

Despite a large dataset (more than 20,000 and 8000 individuals measured for anchovy and sardine respectively) which allowed us to calculate precise estimates of phenotypic differences in L, Gc and their variances, the statistical power in this study is limited by the number of cohorts used (19 cohorts). This lack of statistical power partly explains the large confidence intervals of our parameter estimates but these were particularly robust as there was little variation in these estimates following Jackknifing. Moreover, it is surprising that growth parameters were largely independent of the amount of food in the Bay of Biscay. This might stem from our use of a food proxy chlorophyll‐a which can only capture the variance in zooplankton quantity. This effect prevents us from analysing finer‐scale differences in chlorophyll‐a concentrations. Proxies for predation were not available either. Thus, we probably missed some key elements of the real environment experienced by anchovies and sardines especially if these species use spatial variations in the pelagic environment to optimize their growth. This is supported by the significant intercepts found in some models (i.e. $\Delta M_{L}$ for sardine), indicating that other environmental variables could play an important role but have not been included in our analyses. Finally, the gene flow between stocks could have dampened the adaptive processes that we tried to measure especially for the sardine. Indeed, the European anchovy stock in the Bay of Biscay is a clearly defined management unit with low emigration and immigration rates (Zarraonaindia et al., 2012; Silva et al., 2014; Huret et al., 2020), and the spatial scale at which this stock's dynamics and drivers can be measured are clearly defined. Therefore, any evolutionary or plastic response to fishing or changes in this area's pelagic ecosystem might be more easily detected in anchovy than for the European sardine for which stock boundaries are still discussed (Caballero‐Huertas et al., 2022; Kasapidis, 2014). In this species, gene flow is substantial across North East Atlantic and individuals' movement in and out of the Bay of Biscay might dampen our ability to identify the causes of any adaptive and plastic responses (Caballero‐Huertas et al., 2022).

## 5 CONCLUSION

We were able to show that in anchovy, there was an adaptive response for growth at age‐0 during the early 2000s in response to the selective disappearance of individuals with rapid growth (itself linked to fishing pressure, Boëns et al., 2021). The fishery was closed from 2005 to 2009, enabling the stock to rapidly recover in terms of biomass, which subsequently remained high due to the relatively low harvest rate of this stock. We have shown here that such a high biomass led to density‐dependent processes that maintained a relatively low growth at age‐0 of this stock. Natural selection may lead to a gradual return to the phenotypic characteristics of the early 2000s, as it is expected to favour fish with rapid growth unless environmental changes (warming of surface waters, reduction in food quality, predation) maintain low growth at age‐0 and/or exert further selection pressure acting against individuals with rapid growth at age‐0. In sardine, the phenotypic differences between generations in means and variances in growth and growth compensation were mainly linked to environmental variables and therefore to phenotypic plasticity though the actual driver of these changes remains elusive. In terms of management, in spite of the relatively low harvest rate of the anchovy's stock of the Bay of Biscay, the range of sizes of landed fish is relatively large. Hence, harvesting might remain selective to some extent in this species because of industries' requirements and/or consumption habits of large anchovies. Therefore, a particular care should be given to avoid targeting large anchovies in this area if we are to recover individuals' growth characteristics of the early 2000s. For sardine, in which the harvest rate increased substantially recently, we found no evidence that the selective disappearance of fish with high growth rate at age‐0 was linked to fishing itself (Boëns et al., 2021) but there are more evidences suggesting that changes in food quantity and/or quality might be driving the declines in individuals' growth of this stock (Véron et al., 2020) in a similar manner than in other areas (Brosset et al., 2015, 2017; Saraux et al., 2019). It is therefore important to now identify precisely these factors, understand the physiological constraints acting on growth in European sardines (Brosset et al., 2021) and adjust the harvest rate according to environmental conditions. Overall, our study shows that the apparent similarity of the declines in size‐at‐age in these two exploited populations with similar trophic levels and ecologies is actually underpinned by fundamentally different mechanisms that should be taken into account in the management of these stocks.

## FUNDING INFORMATION

A doctoral grant  was provided to A. Boëns by Ifremer.

## CONFLICT OF INTEREST STATEMENT

There is no conflict of interest.

## Data Availability

The data are available on the repository Seanoe of Ifremer www.seanoe.org.

## APPENDIX A

**TABLE A1:** Coefficients of the average model for the difference in mean growth at age‐0 for anchovy (ΔBiom, difference in biomasse; $\bar{S}$, mean directional selection; ΔPC1, difference in the PC1). PC1 refers to the first principal component summarizing environmental parameters, chlorophyll‐a being highly correlated with that component (see Boëns et al., 2021). The mean and SD of the parameter estimates obtained following Jackknifing are provided.

	Estimate	Std. error	*z* Value	Pr(>|*z*|)	Mean Jack	SD Jack
(Intercept)	−0.09	0.23	0.36	0.72	−0.09	0.07
ΔBiom	−0.45	0.25	1.65	0.10	−0.45	0.09
$\bar{S}$	0.35	0.26	1.25	0.21	0.35	0.05
ΔPC1	0.26	0.24	1.03	0.30	0.27	0.08

**TABLE A2:** Coefficients of the average model for the difference in variance in growth at age‐0 for anchovy (ΔPC1, difference in the PC1; ΔBiom, difference in biomass). PC1 refers to the first principal component summarizing environmental parameters, chlorophyll‐a being highly correlated to that component (see Boëns et al., 2021). The mean and SD of the parameter estimates obtained following Jackknifing are provided.

	Estimate	Std. error	*z* Value	Pr(>|*z*|)	Mean Jack	SD Jack
(Intercept)	0.07	0.24	0.26	0.80	0.06	0.06
ΔPC1	0.39	0.27	1.33	0.18	0.39	0.11

**TABLE A3:** Coefficients of the average model for the difference in mean growth compensation at age‐1 for anchovy (ΔBiom, difference in biomass). The mean and SD of the parameter estimates obtained following Jackknifing are provided.

	Estimate	Std. error	*z* Value	Pr(>|*z*|)	Mean Jack	SD Jack
(Intercept)	−0.02	0.26	0.06	0.95	−0.02	0.07
ΔBiom	0.31	0.26	1.07	0.29	0.30	0.04

**TABLE A4:** Model coefficients for the difference in variance in growth compensation at age‐1 for anchovy. The mean and SD of the parameter estimates obtained following Jackknifing are provided.

	Estimate	Std. error	*z* Value	Pr(>|*z*|)	Mean Jack	SD Jack
(Intercept)	0.19	0.16	1.20	0.25	0.19	0.04

**TABLE A5:** Coefficients of the average model for the difference in mean growth at age‐0 for sardine (ΔBiom; difference in biomass). The mean and SD of the parameter estimates obtained following Jackknifing are provided.

	Estimate	Std. error	*z* Value	Pr(>|*z*|)	Mean Jack	SD Jack
(Intercept)	−0.22	0.08	−2.69	0.02	−0.22	0.04
ΔBiom	0.24	0.10	2.38	0.03	0.24	0.06

**TABLE A6:** Model coefficients for the difference in variance in growth at age‐0 for sardine. The mean and SD of the parameter estimates obtained following Jackknifing are provided.

	Estimate	Std. error	*z* Value	Pr(>|*z*|)	Mean Jack	SD Jack
(Intercept)	−0.09	0.27	−0.32	0.76	−0.09	0.08

**TABLE A7:** Coefficients of the average model for the difference in mean growth compensation at age‐1 for sardine ($\bar{S}$, mean directional selection; ΔBiom, difference in biomass. The mean and SD of the parameter estimates obtained following Jackknifing are provided.

	Estimate	Std. error	*z* Value	Pr(>|*z*|)	Mean Jack	SD Jack
(Intercept)	0.00	0.24	0.00	1.00	0.00	0.06
$\bar{S}$	0.34	0.24	1.27	0.20	0.33	0.08
ΔBiom	0.27	0.25	1.00	0.32	0.28	0.04

**TABLE A8:** Model coefficient for the difference in variance in growth compensation at age‐1 for sardine. The mean and SD of the parameter estimates obtained following Jackknifing are provided.

	Estimate	Std. error	*z* Value	Pr(>|*z*|)	Mean Jack	SD Jack
(Intercept)	0.00	0.24	0.00	1.00	0.00	0.06
